# Supplementary material for: Addressing Complex Social Problems Using the Lens of Family Violence: Valuable Learning from the First Year of an Interdisciplinary Community of Practice
Source: Int J Environ Res Public Health. 2023 Feb 16;20(4):3501. doi: 10.3390/ijerph20043501 (PMC9967701; doi:10.3390/ijerph20043501)
Supplement: Supplementary file 1 [file ijerph-20-03501-s001.zip › ijerph-2189374-supplementary.pdf]

Supplementary File S1: Five Cycles of Value Creation (a condensed version of the original by Wenger, E., Trayner, B., & de Laat, M. (2011) [1])

Cycle 1. Immediate value: Activities and interactions

Activities and interactions can produce value in and of themselves. They can be fun and inspiring. One can get an answer to a question, a solution to a problem, or help with a challenge. Collective reflection can trigger out of the box thinking and open new perspectives. Participants can cooperate on seeking innovative approaches. Just hearing someone else's story can open one's imagination or reveal a new perspective. And being with others who understand one's challenge can be a relief.

Cycle 2. Potential value: Knowledge capital

Activities and interactions can produce 'knowledge capital' whose value lies in its potential to be realized later.

- **Personal assets (human capital).** A useful skill, a key piece of information, or a new perspective; new ideas to address a class of problems; inspiration, caring, confidence, and status; a reawakened … sense of calling and professional identity
- **Relationships and connections (social capital**). …social relations and connections are a form of knowledge capital. The ability to ask questions because one knows who to ask and who to trust; one's reputation is another social achievement that can become a knowledge resource; shared understanding and a common language; potential opportunities for collaboration and the ability to promote a cause; the value of a sense of companionship in the face of demanding tasks and learning challenges.
- **Resources (tangible capital**). Privileged access to certain resources such as information, documents, tools and procedures; networked information sources, tag clouds, mind maps, links and references, search capabilities, visualization tools, and other socio-informational structures that facilitate access to information.
- **Collective intangible assets (reputational capital).** … the reputation of the community or network, the status of a profession, or the recognition of the strategic relevance of the domain; the collective voice or recognition that it provides individuals; increased potential for collective action.
- **Transformed ability to learn (learning capital**): The act of participating in a facilitated network or a community as a valuable way of learning can be enlightening for people for whom formal teaching or training methods have always been seen as the only way to learn. When members have experienced significant learning in networks or communities they can transfer this experience to other contexts.

Cycle 3. Applied value: Changes in practice

Identifying the ways practice has changed in the process of adapting and applying knowledge capital to a specific situation. For instance, reusing a lesson plan or a piece of code, exploiting synergy between business units, changing a procedure, implementing an idea, trying a suggestion, enlisting members of one's network for a cause, or leveraging a collective voice to make a case for an organizational decision, leading to changes or innovations in actions, practice, tools, approaches, or organizational systems.

Cycle 4. Realized value: Performance improvement

The application of new ideas to practice or the use of resources from the community/network is not guaranteed to result in improvements in performance. It is therefore important to reflect on what effects the application of knowledge capital is having on the achievement of what matters to stakeholders, including members who apply a new practice.

Cycle 5. Reframing value: Redefining success

The last cycle of value creation is achieved when social learning causes a reconsideration of the learning imperatives and the criteria by which success is defined, including reframing strategies, goals, as well as values; proposing new metrics for performance that reflect the new definition of success. This redefinition of success can happen at individual, collective, and organizational levels. …It may also mean transforming or leaving behind the existing structure and using this new definition of success to create a new framework.
